# Real-world adoption and use of a ventilation feedback device during out-of-hospital cardiac arrest: a population-based observational study from a French prehospital system

**DOI:** 10.1016/j.resplu.2026.101465

**Published:** 2026-08-25

**Authors:** Jean-Baptiste Pretalli, Marie Da Cunha, Saïd Hachimi-Idrissi, Laure-Estelle Piller, Abdo Khoury

**Affiliations:** a INSERM CIC 1431, Centre d’Investigation Clinique, University Hospital of Besançon, Besançon, France; b Department of Emergency Medicine and Critical Care, Besançon University Hospital, Boulevard Fleming, 25030 Besançon, France; c Université Marie et Louis Pasteur, CHU Besançon, Inserm CIC 1431, SINERGIES (UR4662), F-25000 Besançon, France; d Department of Emergency Medicine, Université Marie et Louis Pasteur, Besançon, France; e Department of Emergency Medicine, Ghent University Hospital, Ghent, Belgium; f Faculty of Medicine and Health Sciences, Ghent University, Ghent; g Faculty of Medicine and Pharmacy, Vrije Universiteit Brussel, Brussels, Belgium; h Doubs Departmental Fire and Rescue Service, Besançon, France

**Keywords:** Implementation, Cardiopulmonary ventilation, Medical device, Out of hospital cardiac arrest, Prehospital medicine, Ventilation feedback devices

## Abstract

**Background:**

Ventilation quality during cardiopulmonary resuscitation remains highly variable despite clear guidelines. Ventilation feedback devices have shown substantial improvements in simulated settings, but real-world implementation data remain limited.

We aimed to evaluate real-world adoption and use of a ventilation feedback device during out-of-hospital cardiac arrest (OHCA) management in a French prehospital system.

**Methods:**

We conducted a retrospective, population-based observational study of adult OHCAs jointly managed by firefighters and Mobile Intensive Care Unit teams in the Doubs region. Cases from 2022 (pre-implementation) and 2024 (post-implementation) were analysed. The primary outcome was device utilisation. Secondary outcomes included return of spontaneous circulation, survival, and neurological status, reported descriptively and exploratorily.

**Results:**

Among 756 patients, 190 (2022) and 166 (2024) met the inclusion criteria. Despite a protocol mandating its systematic use, the device was used in 48/166 (28.9%) cases.

Baseline characteristics and resuscitation variables were comparable between groups. Exploratory analyses showed return of spontaneous circulation in 25.0% with device versus 16.1% without (unadjusted odds ratio (OR) = 1.74 confidence interval (CI) 95% [0.77; 3.93]), and survival at day 30 in 10.4% versus 4.2% (unadjusted OR = 2.63 [0.72; 9.53]). Favourable neurological outcome at day 30 was observed in 10.4% versus 3.4% (unadjusted OR = 3.28 [0.84; 12.8]). Given the observational design and limited sample size, these findings should be interpreted as hypothesis-generating.

**Conclusion:**

This study highlights a discrepancy between protocol and real-world use of a ventilation feedback device during out-of-hospital cardiac arrest. They underscore the need to better understand implementation factors to inform future studies evaluating clinical effectiveness. Future research should incorporate robust implementation strategies and standardised ventilation parameters.

## Highlights

• First real-world description of the ventilation feedback device EOlife®.
• Real-world Ventilation Feedback Device (VFD) usage in prehospital settings reveals implementation challenges.
• Uptake indicates feasibility in out-of-hospital cardiac arrest management.
• Real-world data may inform the design of future clinical efficacy trials of VFDs.

## Introduction

Out-of-hospital cardiac arrest (OHCA) remains a major public health challenge worldwide. In France, incidence is 61.5 cases per 100,000 inhabitants, corresponding to 46,000 OHCA annually. The overall 30-day survival rate remains low at 4.9%. These findings are consistent with epidemiological data reported across other European countries.^1^ According to the latest International Liaison Committee on Resuscitation (ILCOR) Research and Registries Committee report, survival after EMS-treated OHCA continues to vary substantially across participating registries, highlighting marked international heterogeneity in outcomes.^2^

Among survivors, neurological outcomes are frequently poor^3^, ^4^ and OHCA remains among the top three causes of preventable years of life lost in developed countries.^5^, ^6^

Effective management relies on early and high-quality cardiopulmonary resuscitation (CPR). CPR quality depends on both effective chest compressions to restore circulation and effective ventilation to ensure tissue oxygenation.^7^

While decades of research have largely focused on optimising chest compressions,^8^, ^9^, ^10^ ventilation has received less attention, mainly due to technical limitations. Yet, effective ventilation is crucial to maintain gas exchange and prevent hypoxia during prolonged resuscitation, as compression-only CPR cannot provide adequate alveolar ventilation during prehospital care.^11^, ^12^, ^13^, ^14^, ^15^

Despite well-defined international guidelines,^16^, ^17^, ^18^ delivering ventilation according to recommendations remains challenging, regardless of the level or amount of training received. This issue has been recognised for decades^19^ and, despite advances in resuscitation science, remains unresolved. Manikin studies continue to show poor adherence, with only 2.8–22% of ventilations meeting both rate and volume targets.^20^, ^21^, ^22^ Even when considering individual parameters, adherence is limited, for example 22.7% of ventilations meeting volume targets and 51% meeting rate targets.^23^ Real-world data reflect these findings.^24^, ^25^

Various medical devices (MDs) have been developed to assist manual ventilation during OHCA, including metronomes^26^ and impedance threshold devices.^27^, ^28^ More recently, ventilation feedback devices (VFDs) have been introduced. They provide real-time feedback on ventilation rate (VR) and tidal volume (VT).

Manikin studies suggest that VFDs substantially improve ventilation quality. In a previous study, Khoury et al observed an increase in effective ventilation from 15% to 90% with a VFD.^29^ Lyngby et al. reported an increase from 22.1% to 75.3%,^21^ and Charlton et al. found that the proportion of clinicians achieving guideline ventilation ≥50% of the time increased from 9% to 91%.^23^

However, clinical evidence remains limited, partly because evaluating MDs poses inherent challenges. Unlike drugs, whose effects are largely determined by pharmacological properties, their use and effectiveness depend not only on their design but also on user skills, training, environmental conditions, and contextual factors such as workload, stress, fatigue, and noise. Learning curves^30^ and organisational barriers^31^ can also constrain their adoption and effectiveness.

Real-world observational data are therefore needed to assess and optimise VFDs. Such data are also crucial to inform future clinical studies. We aimed primarily to describe the real-word adoption and use of a VFD by firefighters and prehospital Mobile Intensive Care Unit (MICU) teams in a French region. A secondary objective was to explore associations with clinical outcomes to generate hypotheses for future research.

## Methods

### Study design and setting

This single-centre, retrospective, population-based observational study used data only and included all OHCAs in the Doubs region. The study was classified as outside the scope of the French Jardé law. Consequently, no ethics committee approval was required. The study complied with the Declaration of Helsinki and applicable French regulations.

### System of care

The Doubs department includes rural and urban areas (5233 km^2^; 550,000 inhabitants). Approximately 200 OHCAs are managed annually by MICU teams according to the French Cardiac Arrest Registry (RéAC). Patients achieving return of spontaneous circulation (ROSC) may be transported to any of 10 regional hospitals, although the tertiary referral centre is the primary destination.

Emergency calls are received by either the fire service or the emergency medical dispatch centre. A physician-led MICU is dispatched when the dispatch physician considers advanced life support (ALS) appropriate. Firefighters or, less frequently, private ambulance crews without a VFD, provide basic life support (BLS), while the MICU provides ALS and decides whether to continue or terminate resuscitation. Accordingly, only OHCAs managed jointly by firefighters and the MICU were included. Patients receiving ongoing ALS undergo endotracheal intubation.

Since January 2023, all firefighter vehicles have been equipped with the EOlife® VFD.

### Ventilation feedback device

The EOlife® is CE-marked and positioned between the bag-valve device and airway interface. It provides real-time visual feedback on VR and VT through numerical values and graphical indicators, without audible prompts.

For EOlife®, tidal volume (VT) is defined as the volume of gas reaching the lungs during each insufflation, taking into account inspiratory and expiratory flow and air leakage.^32^ The device has no specific contraindications.

Firefighters received standardised training with 3-monthly refreshers and were instructed to use the device for all CPR attempts.

### Study population

All consecutive adult OHCAs jointly managed by firefighters and the MICU between 01/01/2022 and 31/12/2022 (pre-implementation) and 01/01/2024 and 31/12/2024 (post-implementation) were screened.

Patients aged <18 years, pregnant women, women in labour, and vulnerable adults unable to provide consent under French regulations were excluded. Surviving patients were informed about the study when clinically appropriate and could object to data use.

No sample size calculation was performed because this was an exploratory observational study.

### Outcomes

Primary objective: to describe the real-world adoption and use of the VFD by calculating the proportion of eligible OHCA cases in 2024 in which the VFD was used.

Secondary objectives:

• For cases with versus without VFD use in 2024, to describe:
  o OHCA characteristics.
  o CPR characteristics.
  o Aetiologies.
• Explore associations between VFD use and clinical outcomes among OHCA cases managed in 2024:
  o Return of spontaneous circulation (ROSC) in the prehospital setting, at hospital arrival, and at day 30 (or at ICU discharge).
  o The proportion of patients with favourable neurological outcomes (cerebral performance category (CPC) score ≤2) assessed at day 1, and day 30 (or at ICU discharge).
• Assess the temporal comparability of patient and resuscitation characteristics between 2022 and 2024 by comparing OHCA cases managed without VFD use.

Outcome data (ROSC, survival, CPC) were retrospectively extracted from prehospital and hospital records until day 30.

### Statistical analysis

Continuous variables are presented as means ± standard deviations and categorical variables as counts and percentages. Given the observational design, all analyses were exploratory and unadjusted for confounders. Groups were compared using Pearson's chi-squared or Fisher's exact test for categorical variables, and Student's *t*-test or the Wilcoxon-Mann-Whitney test for continuous variables, as appropriate. Normality was assessed using the Shapiro-Wilk test.

Unadjusted odds ratios (ORs) with 95% confidence intervals (CIs) were calculated for ROSC, survival and neurological outcome.

Analyses were performed using SPSS (PASW Statistics version 18.0.0), with *p* < 0.05 considered statistically significant.

Missing data were not imputed. Analyses used available cases for each variable.

## Results

**Fig. 1:**
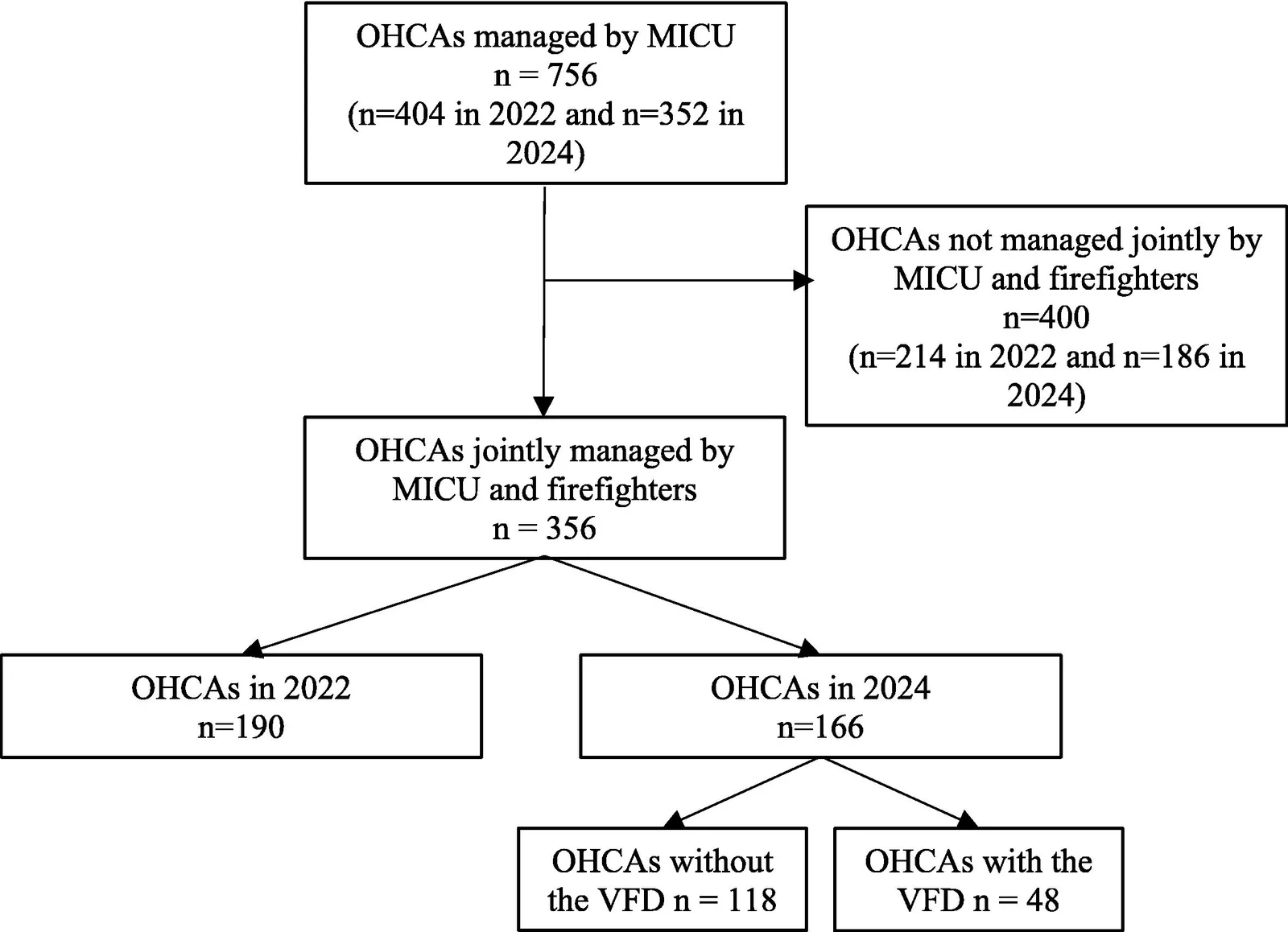
**Inclusion flowchart**. *Abbreviations:* OHCAs, out-of-hospital cardiac arrest; MICU, mobile intensive care unit; VFD, ventilation feedback device.

The MICU managed 404 OHCAs in 2022 and 352 in 2024 (*n* = 756). Among these, 190 in 2022 and 166 in 2024 were managed jointly with firefighters (Fig. 1).

In 2024, 48 (28.9%) OHCAs were managed with the VFD, and 118 (71.1%) without (Table 1). No significant differences were observed between the groups for any of the analysed variables. Patients were predominantly male (71.7%), with a mean age of 67 ± 16.7 years. OHCAs occurred mainly at home (80.1%), with 18.1% in public places and 1.8% in healthcare facilities. Nearly half were witnessed by a bystander (39.1%), and 38.7% had an initial shockable rhythm.

**Table 1:** Out-of-hospital cardiac arrests characteristics.

	**With VFD** ***n* = 48**	**Without VFD** ***n* = 118**	**Total** ***n* = 166**	***p*-value**
Age (years) – mean (SD)	68.1 (15.4)	66.5 (17.2)	67.0 (16.7)	0.627
**Sex – *n* (%)**				
Female	11 (22.9%)	36 (30.5%)	47 (28.3%)	0.385
Male	37 (77.1%)	82 (69.5%)	119 (71.7%)	
Total	48 (100.0%)	118 (100.0%)	166 (100.0%)	
No flow duration (min) – mean (SD)	8.6 (12.8)	7.3 (10.1)	7.7 (11.0)	0.893
**No flow – *n* (%)**				
<5 min	12 (46.2%)	27 (49.1%)	39 (48.1%)	0.805
≥5 min	14 (53.8%)	28 (50.9%)	42 (51.9%)	
Total	26 (100.0%)	55 (100.0%)	81 (100.0%)	
Low flow duration (min) – mean (SD)	28.2 (10.9)	27.3 (15.0)	27.6 (13.6)	0.776
**Shockable rhythm – *n* (%)**				
No	26 (70.3%)	39 (56.5%)	65 (61.3%)	0.166
Yes	11 (29.7%)	30 (43.5%)	41 (38.7%)	
Total	37 (100.0%)	69 (100.0%)	106 (100.0%)	
**Location of OHCA – *n* (%)**				
Home	41 (85.4%)	92 (78.0%)	133 (80.1%)	0.547
Public place	7 (14.6%)	23 (19.5%)	30 (18.1%)	
Healthcare facility	0 (0.0%)	3 (2.5%)	3 (1.8%)	
Total	48 (100.0%)	118 (100.0%)	166 (100.0%)	
**Bystander-witnessed cardiac arrest – *n* (%)**				
No	29 (61.7%)	69 (60.5%)	98 (60.9%)	0.889
Yes	18 (38.3%)	45 (39.5%)	63 (39.1%)	
Total	47 (100.0%)	114 (100.0%)	161 (100.0%)	
**Aetiology – *n* (%)**				
Hypoxic	6 (24.0%)	15 (26.3%)	21 (25.6%)	NA
Cardiovascular	7 (28.0%)	12 (21.1%)	19 (23.2%)	
Palliative	1 (4.0%)	2 (3.5%)	3 (3.7%)	
Suicide (intoxication by medication/defenestration)	1 (4.0%)	4 (7.0%)	5 (6.1%)	
Hanging	5 (20.0%)	10 (17.5%)	15 (18.3%)	
Haemorrhagic	1 (4.0%)	3 (5.3%)	4 (4.9%)	
Weapon-related	0 (0.0%)	1 (1.8%)	1 (1.2%)	
Traumatic	3 (12.0%)	6 (10.5%)	9 (11.0%)	
Septic	0 (0.0%)	1 (1.8%)	1 (1.2%)	
Neurological	1 (4.0%)	1 (1.8%)	2 (2.4%)	
Toxic	0 (0.0%)	2 (3.5%)	2 (2.4%)	
Total	25 (100.0%)	57 (100.0%)	82 (100.0%)	

*Abbreviations:* OHCA, out-of-hospital cardiac arrest. SD, standard deviation VFD, ventilation feedback device.

Missing data: the denominator (*n*) is reported for each variable because the amount of missing data differed across variables.

For “No flow”, 22 (45.8%) data were missing in the group with VFD and 63 (53.4%) in the group without VFD.

For low flow duration, 16 (33.3%) data were missing in the group with VFD and 60 (50.8%) in the group without VFD.

Table 2 compares CPR characteristics in 2024. Only the number of shocks differed significantly between groups. Bystanders initiated CPR in 36.1% of cases, and 34.4% of these were CPR-trained. MICU teams did not perform CPR in nearly half of cases (47.3%).

**Table 2:** Cardiopulmonary resuscitation characteristics.

	**With VFD** ***n* = 48**	**Without VFD** ***n* = 118**	**Total** ***n* = 166**	***p*-value**
**Bystander CPR – *n* (%)**				
No	13 (31.0%)	39 (38.2%)	52 (36.1%)	0.408
Yes	29 (69.0%)	63 (61.8%)	92 (63.9%)	
Total	42 (100.0%)	102 (100.0%)	144 (100.0%)	
**CPR by trained bystander – *n* (%)**				
No	2 (20.0%)	9 (40.9%)	11 (34.4%)	0.425
Yes	8 (80.0%)	13 (59.1%)	21 (65.6%)	
Total	10 (100.0%)	22 (100.0%)	32 (100.0%)	
**CPR provided by MICU – *n* (%)**				
No	18 (38.3%)	60 (50.8%)	78 (47.3%)	0.145
Yes	29 (61.7%)	58 (49.2%)	87 (52.7%)	
Total	47 (100.0%)	118 (100.0%)	165 (100.0%)	
**Epinephrine dose – *n* (%)**				
<5 mg	18 (64.3%)	36 (63.2%)	54 (63.5%)	0.919
≥5 mg	10 (35.7%)	21 (36.8%)	31 (36.5%)	
Total	28 (100.0%)	57 (100.0%)	85 (100.0%)	
**Amiodarone administered – *n* (%)**				
No	47 (97.9%)	106 (89.8%)	153 (92.2%)	0.111
Yes	1 (2.1%)	12 (10.2%)	13 (7.8%)	
Total	48 (100.0%)	118 (100.0%)	166 (100.0%)	
**Number of shocks delivered – *n* (%)**				
<5	10 (100.0%)	20 (66.7%)	30 (75.0%)	0.043
≥5	0 (0.0%)	10 (33.3%)	10 (25.0%)	
Total	10 (100.0%)	30 (100.0%)	40 (100.0%)	
**EtCO_2_ at intubation – *n* (%)**				
≤10 mmHg	0 (0.0%)	1 (2.3%)	1 (1.5%)	1
>10 mmHg	24 (100.0%)	42 (97.7%)	66 (98.5%)	
Total	24 (100.0%)	43 (100.0%)	67 (100.0%)	
**LUCAS device used – *n* (%)**				
No	3 (27.3%)	5 (21.7%)	8 (23.5%)	1
Yes	8 (72.7%)	18 (78.3%)	26 (76.5%)	
Total	11 (100.0%)	23 (100.0%)	34 (100.0%)	

*Abbreviations:* CPR, cardiopulmonary resuscitation. OHCA, out-of-hospital cardiac arrest. SD, standard deviation VFD, ventilation feedback device. LUCAS, mechanical chest compression device used.

Missing data: the denominator (*n*) is reported for each variable because the amount of missing data differed across variables.

Regarding OHCAs managed in 2024, clinical outcomes, including ROSC, day-1 survival, day-30 survival, and favourable neurological outcome, are presented in Table 3.

**Table 3:** Outcomes and aetiologies.

	**With VFD** ***n* = 48**	**Without VFD** ***n* = 118**	**Total** ***n* = 166**	**Unadjusted odds ratio** **(CI 95%)**	***p*-value**
**ROSC – *n* (%)**					
No	36 (75.0%)	99 (83.9%)	135 (81.3%)	1.74 [0.77; 3.93]	0.182
Yes	12 (25.0%)	19 (16.1%)	31 (18.7%)		
Total	48 (100.0%)	118 (100.0%)	166 (100.0%)		
**Survival at day 1 – *n* (%)**					
No	40 (83.3%)	108 (91.5%)	148 (89.2%)	2.16 [0.80; 5.86]	0.124
Yes	8 (16.7%)	10 (8.5%)	18 (10.8%)		
Total	48 (100.0%)	118 (100.0%)	166 (100.0%)		
**Survival at day 30 – *n* (%)**					
No	43 (89.6%)	113 (95.8%)	156 (94.0%)	2.63 [0.72; 9.53]	0.155
Yes	5 (10.4%)	5 (4.2%)	10 (6.0%)		
Total	48 (100.0%)	118 (100.0%)	166 (100.0%)		
**Neurological status at day 30 – *n* (%)**					
CPC = 1	5 (10.4%)	4 (3.4%)	9 (5.4%)	3.28 [0.84; 12.8]	0.123
CPC ≠ 1	43 (89.6%)	113 (96.6%)	156 (94.5%)		
Total	48 (100.0%)	117 (100.0%)	165 (100.0%)		
**Aetiology – *n* (%)**					
Post-anoxic encephalopathy	2 (50.0%)	4 (50.0%)	6 (50.0%)	Not applicable	0.697
Multiorgan failure	1 (25.0%)	2 (25.0%)	3 (25.0%)		
New cardiac arrest	0 (0.0%)	2 (25.0%)	2 (16.7%)		
Hypoxic cause	1 (25.0%)	0 (0.0%)	1 (8.3%)		
Total	4 (100.0%)	8 (100.0%)	12 (100.0%)		

*Abbreviations:* CPC score, cerebral performance category score. OHCA, out-of-hospital cardiac arrest. ROSC, return of spontaneous circulation. VFD, ventilation feedback device.

Unadjusted ORs represent the odds of ROSC, survival, or CPC = 1, respectively, with VFD versus without.

Missing data: the denominator (*n*) is reported for each variable because the amount of missing data differed across variables.

Table 4 presents those outcomes for OHCAs managed without VFD in 2022.

**Table 4:** Outcomes and aetiologies for OHCAs managed without VFD in 2022 and 2024.

	**OHCAs in 2022** ***n* = 190**	**OHCAs in 2024** ***n* = 118**	**Total** ***n* = 308**	**Unadjusted odds ratio** **(CI 95%)**	***p*-value**
**ROSC – *n* (%)**					
No	157 (82.6%)	99 (83.9%)	256 (83.1%)	0.913	0.773
Yes	33 (17.4%)	19 (16.1%)	52 (16.9%)	[0.49; 1.69]	
Total	190 (100.0%)	118 (100.0%)	308 (100.0%)		
**Survival at day 1 – *n* (%)**					
No	166 (87.4%)	108 (91.5%)	274 (89.0%)	0.640	0.258
Yes	24 (12.6%)	10 (8.5%)	34 (11.0%)	[0.29; 1.39]	
Total	190 (100.0%)	118 (100.0%)	308 (100.0%)		
**Survival at day 30 – *n* (%)**					
No	181 (95.3%)	113 (95.8%)	294 (95.5%)	0.89	0.838
Yes	9 (4.7%)	5 (4.2%)	14 (4.5%)	[0.29; 2.72]	
Total	190 (100.0%)	118 (100.0%)	308 (100.0%)		
**Neurological status at day 30 – *n* (%)**					
CPC = 1	6 (3.2%)	4 (3.4%)	10 (3.3%)	1.08	1
CPC ≠ 1	184 (96.8%)	113 (96.6%)	297 (96.7%)	[0.30; 3.94]	
Total	190 (100.0%)	117 (100.0%)	307 (100.0%)		
**Cause of death – *n* (%)**					
Post-anoxic encephalopathy	10 (5.3%)	4 (3.4%)	14 (4.5%)		
Multiorgan failure	5 (2.6%)	2 (1.7%)	7 (2.3%)		
New cardiac arrest	4 (2.1%)	2 (1.7%)	6 (1.9%)		
Hypoxic cause	0 (0.0%)	0 (0.0%)	0 (0.0%)		
Total	19 (100.0%)	8 (100.0%)	27 (100.0%)		

*Abbreviations:* CPC score, cerebral performance category score. OHCA, out-of-hospital cardiac arrest. ROSC. return of spontaneous circulation.

Unadjusted ORs represent the odds of ROSC, 1-day survival, 30-day survival, or CPC = 1 in 2024 versus 2022.

Missing data: the denominator (*n*) is reported for each variable because the amount of missing data differed across variables.

Although the VFD provides real-time feedback, objective ventilation data (VT, VR, and leak magnitude) were not recorded, preventing assessment of ventilation quality and guideline adherence and limiting interpretation of its relationship with clinical outcomes.

## Discussion

This study provides an evaluation of VFD adoption and real-world use within a mature prehospital system, revealing a marked gap between recommended and actual use. Among 166 consecutive OHCAs included in 2024, the VFD was used in only 48 cases (28.9%). These findings underscore that the implementation of VFDs is shaped by multifactorial barriers that must be addressed in the design of future clinical efficacy studies to ensure both feasibility and methodological robustness. In keeping with established evidence on the adoption of novel technologies in emergency and prehospital care, this gradual uptake likely reflects a complex interplay between human factors, training exposure, operational workload, and broader system-level constraints.

Importantly, this study was designed as an adoption and real-world use focused evaluation rather than a clinical effectiveness trial. From this perspective, the observed utilisation rate is a key outcome, reflecting the real-world integration of the device into clinical workflows.

Interpreting the observed utilisation rate is challenging, as no clear benchmarks exist for the adoption of MDs. According to Rogers' diffusion of innovations theory, new technology adoption typically follows an S-shaped curve, with slow initial uptake before wider dissemination as familiarity, confidence, and organisational support increase.^33^ The utilisation rate observed here may reflect the early adoption phase, although longitudinal data are required to determine the actual position on the adoption curve.^34^, ^35^

Even in randomised OHCA studies, full adherence to the planned intervention is rare. For example, in a randomised trial of the LUCAS-2 device, only 60% of patients received mechanical CPR,^36^ despite the close protocol monitoring typically associated with randomised trials.^37^ In real-world settings, the use of mechanical CPR devices is more limited (<10%) with only modest increases over time.^38^ Although mechanical CPR devices are indicated only in selected clinical situations and therefore differ from VFDs, this example illustrates the challenges of achieving complete adherence to device-based interventions during OHCA management.

Identifying and addressing barriers to implementation is critical to ensure adequate adoption and valid evaluation. Failure to anticipate them may impede participant recruitment, compromise study feasibility, and bias assessment of the device’s effectiveness.

Incomplete adoption results from the interaction of multiple determinants, at the device, user, environmental, and organisational levels.

Regarding the device, deployment appeared feasible in a wide range of prehospital situations. No statistically significant differences in patient or scene characteristics were observed between CPR with or without the VFD. This suggests that obvious contraindications or systematic operational constraints are unlikely to explain the utilisation rate. However, ergonomic barriers or operational barriers were not formally assessed. With respect to training, previous simulation-based studies indicated that the EOlife® VFD could be learned and operated rapidly.^29^ Nevertheless, despite implementation one year earlier and standardised training with refreshers, some users may not have been trained, especially due to staff turnover.

Skill transfer from simulation to real-life resuscitation is complex. Adoption of new technologies is often slow, even when they have the potential to improve patient outcomes. This phenomenon is particularly pronounced in emergency medicine,^39^ and even more so in prehospital settings where environments are challenging and uncontrolled.^40^ Real-time feedback devices for ventilation or chest compressions can improve CPR quality but also increase the substantial cognitive load associated with CPR, which may hinder adoption.^41^ In addition, behavioural responses and beliefs, such as avoidance or delayed initiation, rank among the top four factors influencing the implementation of new technologies and should be taken into account.^40^ In prehospital settings with limited resources and challenging conditions, introducing new tools requires a careful, progressive implementation strategy.

Organisational factors such as system context, resource availability, and communication may also influence adoption of new practices and technologies.^40^

These factors likely contributed to the utilisation rate, despite prior implementation and training efforts.

Regarding the exploratory analysis, our observations are encouraging, although uncertainty remains due to the wide confidence intervals. These findings justify prospective studies. In 2023, Idris et al., assessing ventilation quality using thoracic bioimpedance, reported a significant association between higher-quality ventilation and clinical outcomes in 1976 OHCA cases (survival to hospital discharge of 13.5% versus 4.1% (*p* < 0.0001), and survival with favourable neurological outcome of 10.6% versus 2.4% (*p* < 0.0001)).^42^ In our study, we did not have access to ventilation parameters and therefore could not assess the relationship between VFD use and ventilation quality.

Future studies should aim to simultaneously assess VFD use, ventilation quality, and clinical outcomes, as this represents a key methodological challenge. In a before-and-after study including 412 patients conducted in 2024, Drennan et al. observed an improvement in the proportion of ventilation within recommended targets. However, they found no corresponding improvement in patient outcomes.^24^

This challenge may partly reflect the lack of standardised definitions of ventilation quality, usually based on VT and VR but varying across studies. Some considered both parameters, whereas others focus on only one.^21^, ^23^, ^29^ In addition, VT may be reported with or without accounting for mask leakage and is sometimes incorrectly equated with insufflated volume, potentially altering the estimation of effective ventilation.

Similarly, the definition of hyperventilation remains unclear. Hyperventilation is frequently reported in manikin^21^, ^23^, ^29^ and in clinical studies,^43^, ^44^, ^45^ with several associated factors^46^ and physiological consequences.^18^, ^43^, ^47^, ^48^, ^49^, ^50^

However, hyperventilation is not consistently associated with poorer clinical outcomes. In a retrospective study of 337 adults resuscitated in the prehospital setting, hyperventilation occurred in 85.2% of cases, but was not associated with ROSC, survival to hospital discharge, or one-year survival with favourable neurological outcome.^51^

Several mechanisms may explain this discrepancy. First, reduced lung compliance and alveolar collapse during CPR have been reported in animal and clinical studies,^52^, ^53^, ^54^ potentially influencing the relationship between delivered and effective ventilation.

Technical factors should also be considered. In a prospective observational study examining ventilation during 30:2 CPR by BLS teams, median leakage was 222 [160–261] mL.^55^ Such leaks may partially reduce effective alveolar ventilation, suggesting a distinction between measured hyperventilation and effective alveolar ventilation.

Finally, as current guidelines are largely based on observational studies and expert consensus, thresholds for VT and VR remain poorly defined. Evidence that closer adherence to these targets directly improves patient outcomes remains limited. Accordingly, ventilation quality, defined here as adherence to recommended VT and VR, should be considered an intermediate process outcome rather than a validated surrogate for survival or favourable neurological recovery. Future studies should not only evaluate whether VFDs improve adherence to recommended ventilation targets, but also whether alternative thresholds or definitions of optimal ventilation are more strongly associated with patient-centred outcomes. Some VFDs can also record ventilation parameters, which may help refine ventilation targets.^56^

Medical devices face barriers to implementation, and their performance depends not only on their intrinsic properties but also on user skills, training, and care context, with additional constraints in time-critical settings such as OHCA.

In this study, the uptake suggests that integration of the VFD into routine OHCA management is feasible. This may partly reflect both favourable device-related characteristics, such as ease of use and limited logistical burden, and growing awareness of the importance of ventilation quality during resuscitation.

Future studies should incorporate robust implementation strategies alongside standardised assessment of ventilation quality. They may include providing initial and refresher training, monitoring of device utilisation and reasons for non-use, assessment of user experience and perceived barriers, regular feedback to participating teams, and collection of ventilation quality data. Such an approach would help distinguish implementation failure (device not used as intended), performance failure (device does not improve ventilation quality), and true lack of clinical effectiveness, recognising that absence of clinical benefit may reflect inadequate implementation, suboptimal adherence, or uncertainty regarding the ventilation targets that the device is designed to achieve.

### Strengths and limitations

The population-based design and consecutive inclusion within a well-defined prehospital system provide a real-world assessment of VFD adoption under routine operational conditions and reduce selection bias. By focusing on adoption and actual use rather than clinical effectiveness, the study provides complementary information for VFD evaluation and implementation.

Nevertheless, exploratory outcome analyses were unadjusted for confounders. Given the limited sample size and observational design, these analyses were not powered for formal inference and should be considered hypothesis-generating.

The limited sample size also increases the risk of type II error, and clinically relevant differences may have been missed. VFD use was not randomly assigned and may therefore reflect underlying differences in provider characteristics rather than a true device effect. Firefighters who utilised the VFD may have demonstrated higher protocol adherence, more recent training exposure, or greater engagement with high-quality CPR delivery. Patients receiving the VFD tended to have characteristics associated with a better prognosis, suggesting preferential use in patients perceived as more likely to achieve successful resuscitation. This selection bias is inherent to observational evaluations of device adoption in routine practice and highlights the need for complementary randomised controlled evaluations to establish causal inference. Despite these limitations, survival rates in the non-VFD group were consistent with pre-implementation rates in 2022, with no major changes in prehospital practice over the study period (Table 4; Supplementary Tables 1 and 2), suggesting that temporal changes in the prehospital system are unlikely to fully explain the findings. However, residual confounding and unmeasured between-group differences remain possible.

VT and VR were not recorded in routine practice by firefighters. This major limitation prevented the assessment of guideline adherence. CPR duration, response times, other prehospital interventions and provider characteristics could not be evaluated due to limited retrospective data. User perceptions, device usability, and training coverage were also not formally assessed, which may have influenced adoption and effectiveness.

## Conclusion

This study highlights a substantial gap between protocol recommendations and real-world VFD use during OHCA. This gap likely reflects interacting device-, user-, environmental-, and organisational-level determinants. VFD evaluation should consider the entire pathway from adoption to clinical effectiveness, using standardised ventilation parameters and prospective assessment of ventilation quality and clinical outcomes.

## CRediT authorship contribution statement

**Jean-Baptiste Pretalli:** Writing – review & editing, Writing – original draft, Methodology, Formal analysis, Data curation, Conceptualization. **Marie Da Cunha:** Investigation, Data curation. **Saïd Hachimi-Idrissi:** Writing – review & editing. **Laure-Estelle Piller:** Investigation. **Abdo Khoury:** Writing – review & editing, Supervision, Methodology, Conceptualization.

## Declaration of competing interest

This research did not receive any specific grant from funding agencies in the public, commercial, or not-for-profit sectors.

## Appendix A Supplementary material

Supplementary Fig. 1 Supplementary material includes the STROBE checklist, results on out-of-hospital and cardiopulmonary resuscitation characteristics of OHCAs managed without VFD in 2022 and 2024, and photographs of the EOlife device (Archeon Medical).
