# Supplementary material for: Real-world adoption and use of a ventilation feedback device during out-of-hospital cardiac arrest: a population-based observational study from a French prehospital system
Source: Resusc Plus. 2026 Aug 25;31:101465. doi: 10.1016/j.resplu.2026.101465 (PMC13571132; doi:10.1016/j.resplu.2026.101465)
Supplement: Supplementary file 1 [file mmc1.docx]

Supplementary figure 1 : EOlife - Archeon Medical (personal pictures)

(A) Countdown timer indicating time to the next ventilation.

(B) Volume feedback gauge indicating insufficient (orange), adequate (green), or excessive (red) delivered volume.
