# Supplementary material for: Real-world adoption and use of a ventilation feedback device during out-of-hospital cardiac arrest: a population-based observational study from a French prehospital system
Source: Resusc Plus. 2026 Aug 25;31:101465. doi: 10.1016/j.resplu.2026.101465 (PMC13571132; doi:10.1016/j.resplu.2026.101465)
Supplement: Supplementary file 2 [file mmc2.docx]

Supplementary table 1 : Out-of-hospital characteristics for OHCAs managed without VFD in 2022 and 2024

|  | OHCAs in 2022 n=190 | OHCAs in 2024 n=118 | TOTAL n=308 | p value |
| --- | --- | --- | --- | --- |
| Age (years) - mean (SD) | 66,2 (18,7) | 66,5 (17,2) | 66,3 (18,1) | 0,826 |
| Sex - n (%) |  |  |  |  |
| Female | 66 (34,7%) | 36 (30,5%) | 102 (33,1%) | 0,443 |
| Male | 124 (65,3%) | 82 (69,5%) | 206 (66,9%) |  |
| Total | 190 (100,0%) | 118 (100,0%) | 308 (100,0%) |  |
| No flow duration (minutes) - mean (SD) | 6,9 (7,6) | 7,3 (10,1) | 7,0 (8,6) | 0,982 |
| No flow - n (%) |  |  |  |  |
| < 5 minutes | 43 (45,3%) | 27 (49,1%) | 70 (46,7%) | 0,651 |
| ≥ 5 minutes | 52 (54,7%) | 28 (50,9%) | 80 (53,3%) |  |
| Total | 95 (100,0%) | 55 (100,0%) | 150 (100,0%) |  |
| Low flow duration (minutes) - mean (SD) | 31,3 (16,3) | 27,3 (15,0) | 30,0 (16,0) | 0,153 |
| Shockable rhythm - n (%) |  |  |  |  |
| No | 83 (70,9%) | 39 (56,5%) | 122 (65,6%) | 0,046 |
| Yes | 34 (29,1%) | 30 (43,5%) | 64 (34,4%) |  |
| Total | 117 (100,0%) | 69 (100,0%) | 186 (100,0%) |  |
| Location of OHCA - n (%) |  |  |  |  |
| Home | 149 (78,4%) | 92 (78,0%) | 241 (78,2%) | 0,051 |
| Public place | 25 (13,2%) | 23 (19,5%) | 48 (15,6%) |  |
| Healthcare facility | 16 (8,4%) | 3 (2,5%) | 19 (6,2%) |  |
| Total | 190 (100,0%) | 118 (100,0%) | 308 (100,0%) |  |
| Bystander-witnessed cardiac arrest - n (%) |  |  |  |  |
| No | 108 (58,7%) | 69 (60,5%) | 177 (59,4%) | 0,754 |
| Yes | 76 (41,3%) | 45 (39,5%) | 121 (40,6%) |  |
| Total | 184 (100,0%) | 114 (100,0%) | 298 (100,0%) |  |
| Etiology- n (%) |  |  |  |  |
| Hypoxic | 19 (20,9%) | 15 (26,3%) | 34 (23,0%) |  |
| Cardiovascular | 22 (24,2%) | 12 (21,1%) | 34 (23,0%) |  |
| Palliative | 6 (6,6%) | 2 (3,5%) | 8 (5,4%) |  |
| Suicide (Intoxication by medication/defenestration) | 11 (12,1%) | 4 (7,0%) | 15 (10,1%) |  |
| Hanging | 21 (23,1%) | 10 (17,5%) | 31 (20,9%) |  |
| Hemorrhagic | 5 (5,5%) | 3 (5,3%) | 8 (5,4%) |  |
| Weapon-related | 3 (3,3%) | 1 (1,8%) | 4 (2,7%) |  |
| Traumatic | 3 (3,3%) | 6 (10,5%) | 9 (6,1%) |  |
| Septic | 0 (0,0%) | 1 (1,8%) | 1 (0,7%) |  |
| Neurological | 0 (0,0%) | 1 (1,8%) | 1 (0,7%) |  |
| Toxic | 1 (1,1%) | 2 (3,5%) | 3 (2,0%) |  |
| Total | 91 (100,0%) | 57 (100,0%) | 148 (100,0%) |  |

Abbreviations: OHCA: out-of hospital cardiac arrest. SD. standard deviation.

Missing data: we provided the total for each variable to allow the identification of the missing data.
