# Supplementary material for: Real-world adoption and use of a ventilation feedback device during out-of-hospital cardiac arrest: a population-based observational study from a French prehospital system
Source: Resusc Plus. 2026 Aug 25;31:101465. doi: 10.1016/j.resplu.2026.101465 (PMC13571132; doi:10.1016/j.resplu.2026.101465)
Supplement: Supplementary file 3 [file mmc3.docx]

Supplementary table 2 : cardiopulmonary resuscitation characteristics for OHCAs managed without VFD in 2022 and 2024

|  | OHCAs in 2022 n=190 | OHCAs in 2024 n=118 | TOTAL n=308 | p value |
| --- | --- | --- | --- | --- |
| Bystander CPR - n (%) |  |  |  |  |
| No | 62 (36,9%) | 39 (38,2%) | 101 (37,4%) | 0,827 |
| Yes | 106 (63,1%) | 63 (61,8%) | 169 (62,6%) |  |
| Total | 168 (100,0%) | 102 (100,0%) | 270 (100,0%) |  |
| CPR by trained bystander - n (%) |  |  |  |  |
| No | 8 (24,2%) | 9 (40,9%) | 17 (30,9%) | 0,190 |
| Yes | 25 (75,8%) | 13 (59,1%) | 38 (69,1%) |  |
| Total | 33 (100,0%) | 22 (100,0%) | 55 (100,0%) |  |
| CPR provided by MICU - n (%) |  |  |  |  |
| No | 82 (44,6%) | 60 (50,8%) | 142 (47,0%) | 0,286 |
| Yes | 102 (55,4%) | 58 (49,2%) | 160 (53,0%) |  |
| Total | 184 (100,0%) | 118 (100,0%) | 302 (100,0%) |  |
| Epinephrine dose - n (%) |  |  |  |  |
| < 5 mg | 61 (64,9%) | 36 (63,2%) | 97 (64,2%) | 0,829 |
| ≥ 5 mg | 33 (35,1%) | 21 (36,8%) | 54 (35,8%) |  |
| Total | 94 (100,0%) | 57 (100,0%) | 151 (100,0%) |  |
| Amiodarone administered - n (%) |  |  |  |  |
| No | 176 (92,6%) | 106 (89,8%) | 282 (91,6%) | 0,390 |
| Yes | 14 (7,4%) | 12 (10,2%) | 26 (8,4%) |  |
| Total | 190 (100,0%) | 118 (100,0%) | 308 (100,0%) |  |
| Number of shocks delivered - n (%) |  |  |  |  |
| < 5 | 20 (60,6%) | 20 (66,7%) | 40 (63,5%) | 0,618 |
| ≥ 5 | 13 (39,4%) | 10 (33,3%) | 23 (36,5%) |  |
| Total | 33 (100,0%) | 30 (100,0%) | 63 (100,0%) |  |
| EtCO₂ at intubation - n (%) | ç |  |  |  |
| ≤ 10 mmHg | 14 (16,9%) | 1 (2,3%) | 15 (11,9%) | 0,017 |
| > 10 mmHg | 69 (83,1%) | 42 (97,7%) | 111 (88,1%) |  |
| Total | 83 (100,0%) | 43 (100,0%) | 126 (100,0%) |  |
| LUCAS device used - n (%) |  |  |  |  |
| No | 9 (28,1%) | 5 (21,7%) | 14 (25,5%) | 0,592 |
| Yes | 23 (71,9%) | 18 (78,3%) | 41 (74,5%) |  |
| Total | 32 (100,0%) | 23 (100,0%) | 55 (100,0%) |  |

Abbreviations: OHCA: out-of hospital cardiac arrest. CPR. cardiopulmonary resuscitation SD. standard deviation; LUCAS. mechanical chest compression device used.

Missing data: we provided the total for each variable to allow the identification of the missing data.
